# Supplementary material for: Inspection confirmed mold damage in schools and new use of drugs for airway obstruction: A cohort study
Source: PLoS One. 2025 Oct 8;20(10):e0333486. doi: 10.1371/journal.pone.0333486 (PMC12507237; doi:10.1371/journal.pone.0333486)
Supplement: S1 Text — (DOCX) [file pone.0333486.s001.docx]

**S1. Methods and discussion of exposure assessment**

We had access to an electronic database, where all inspection reports from the primary and secondary school buildings were collected starting from year 2002. Mold damage in the buildings in 2004 was estimated based on retrospective review of all inspection reports done in the buildings between 2002 and end of 2021. That is, reports of the detection of mold damage in a school building between 2005 and 2021 were also considered in the assessment of exposure of students in 2004. This was done because mold damage in Finnish buildings typically develops hidden within structures over a long period of time and stays unnoticed for extended periods of time before confirmed, inspection-based detection. Review of inspection data was done blinded to the health data by one of the authors (T.M.) with long experience in reviewing moisture damage reports. Buildings with insufficient data on mold damage were excluded from the present analyses. All included buildings built before 1995 had inspection reports available. If the building was built after 1995 and had no inspection reports available, it was coded as no damage.

We focused on determining the extent of exposure of the students to mold damage. Mold damage was defined as moisture damage with signs of microbial growth, which may cause harmful exposure to the students. Therefore, we did not consider areas of the buildings, which were not in normal daily use of the students, like storage areas. First it was estimated if the mold damage had been detected in the building by end of 2021 and if it was likely to be present already in 2004. For example, if there had been a water damage after 2004 leading to microbial growth, it was not considered in the classification. On the other hand, gradually developing damage due to a faulty design of the structure was considered to be present already in 2004, even if it was confirmed years later.

Then it was roughly estimated how extensive the exposure likely was in 2004, based on the proportion of damaged area in relation to the total floor area of all stories of the school. ‘Small damage’ refers to single, local damages with a size of at the most 1-2 m^2^. ‘Limited damage’ was defined as local mold damage in several locations, but still potentially relevant to indoor exposure in clearly less than one third of the building’s floor area. ‘Wide damage’ was estimated to have possibly affected more than one third of the building’s floor area and ‘very wide damage’ practically the entire building. Typically, in very wide damage students had been moved to another building to avoid exposure or it had been decided to bring forward the complete renovation of the whole building preceded by temporary measures to reduce exposure, like use of air cleaners. A separate category was defined for buildings demolished due to mold damage. In addition, the year of the report where the extent of damage was described was coded into five-year groups to allow estimating the possible effect of the time of discovering the damage on health.

As hidden mold damage in Finnish public buildings is often found in several structures like base floor and walls^1^, more than two-thirds of the buildings could be fairly easily classified to have wide damage, in comparison to the buildings with no, small or limited damage. Accurate assessment of extent of exposure to mold damage was difficult, if there were several and widely damaged structures. Therefore, buildings were mostly categorized in the most severe class, very wide damage, if there were also other evidence for the severity of the situation, like evacuation of the building or temporary use of air cleaners or the building was demolished due to mold damage. The decision to introduce such temporary measures is influenced by many other factors, like time and resources, and also the concern among the parents, which can sometimes be high in Finland.^2^ However, the health risks in the present study were very similar between the buildings with wide or very wide damage, whereas some suggestion for higher risks were seen in buildings with limited damage.

In the present study, we did not extract data on microbiological determinations from the technical reports, if such were done, as typically mold damage is confirmed by other methods and microbial samples are taken only in a minority of the cases to complement the assessment.^3^ For the same reason our analyses did not consider levels of other indoor air impurities, such as volatile organic compounds.

The method used in the present study to assess mold damage differs from previous prospective studies. Studies done in homes have shown an association between moisture damage and mold and development of new asthma^4-8^, also in Finland^9-11^, but we are not aware of earlier prospective studies on development of asthma including multiple schools, workplaces, or other public buildings.^4-6^^,12^ Most of the past studies have been based on self-reports or short walk-through inspections for visible signs of moisture or mold growth or smell in homes. Visible mold in classrooms is, however, rare in Finnish schools and public buildings.^1,13^ Instead, moisture damage and mold are typically hidden inside the structures of the building, especially in structures with design errors in the construction, so called risk structures.^3,14-15^ Therefore, detailed invasive methods have been developed to assess the extent of such damage. ^14-15^ Before 2010, when little more than half of the inspections of the present study was done, the guidelines in use were similar, but less detailed.^16^ For example, lack or wrong location of waterproof membrane in the load-bearing structures of the building makes them susceptible to moisture rising from the ground. Therefore, like in the technical reports used in the present study, presence of mold damage in public buildings in Finland is assessed by identifying such risk structures first, followed by inspections to confirm damage situation, including opening building structures.^14-15^ Material samples are taken for cultivation if the microbial growth is not obvious by visual or technical inspection. These examinations and interpretation of the findings are currently done according to detailed national guidelines.^3,15^ These guidelines were mainly developed to identify the extent of mold damage and to plan remediations, conservatively assuming a linear relationship between the extent of mold damage and adverse health effects. Given the current lack of knowledge^17^ on the health-relevant characteristics of mold damage, this is a fair assumption, but as such, these guidelines are not tailored to accurately quantify the associated health risks.

Extent and severity of mold damage was determined based on all technical inspections done in the buildings until end of 2021. Although national guidelines for such inspections were already in place since 1990’s, they were better and better followed with time^3,14,16^, but, in contrast to what we initially expected, the year when mold damage was detected was not associated with either risk of new asthma or new use of drugs for airway obstruction. Also, using data from all inspections until 2021 increases the likelihood that problems in the studied schools with mold are detected with repeated inspections or become otherwise apparent, triggering dedicated investigations. Inspections were also done by different companies, which causes uncertainty, but there were several reports on practically all damaged schools and all reports were reviewed and data extracted by a single expert with long experience in reviewing moisture damage reports.

The potential association between mold damage in schools and risk of new asthma could be most accurately assessed, if we had estimates for each students’ personal exposure to mold. This was not possible in the present study, so we had to settle for average exposure and risk in a building. For example, we were not able to take into account the location of the student’s own classroom in relation to mold damage or the effect of time the student spent in the school, as we only had a list of all students in the school buildings in February 2004 and their age, but no data on the location of the class room or the exact year of entering or leaving the school due to e.g., the family changing address. One would expect that children with longer duration of exposure would show stronger associations with asthma compared to students who spent less time in the school building. This was, however, not really supported by the sensitivity analyses excluding students with potentially shorter duration of exposure, i.e. those children whose home address changed during their school years and excluding those four primary schools, where classes were in two different buildings, as the sensitivity analyses showed similar associations.

**References**

1. Marttila T, Lahdensivu J, Pekkanen J. Comprehensive assessment brings out shortcomings in almost every school building. Proceedings of the 12th Nordic Building Physics Conference. 7-9.2020, Tallinn, Estonia. In: E3S Web of Conferences. 172, 23002. <https://doi.org/10.1051/e3sconf/202017223002>

2. Nissilä JJ, Savelieva K, Lampi J, Ung-Lanki S, Elovainio M, Pekkanen J. Parental worry about indoor air quality and student symptom reporting in primary schools with or without indoor air quality problems. Indoor Air 2019; 29(5):865–873.

3. Annila PJ, Lahdensivu J, Suonketo J, Pentti M. Practical Experiences from Several Moisture Performance Assessments. J.M.P.Q. Delgado (ed.), Recent Developments in Building Diagnosis Techniques, Building Pathology and Rehabilitation 2016;5:1-20.

4. World Health Organization. WHO guidelines for indoor air quality: dampness and mould. Regional Office for Europe, Copenhagen, Denmark. WHO, 2009

5. Hurraß J, Heinzow B, Walser-Reichenbach S, Aurbach U, Becker S, Bellmann R, Bergmann KC, Cornely OA, Engelhart S, Fischer G, Gabrio T, Herr CEW, Joest M, Karagiannidis C, Klimek L, Köberle M, Kolk A, Lichtnecker H, Lob-Corzilius T, Mülleneisen N, Nowak D, Rabe U, Raulf M, Steinmann J, Steiß JO, Stemler J, Umpfenbach U, Valtanen K, Werchan B, Willinger B, Wiesmüller GA. AWMF mold guideline "Medical clinical diagnostics for indoor mold exposure" - Update 2023 AWMF Register No. 161/001. Allergol Select. 2024 May 3;8:90-198.

6. Agache I, Canelo-Aybar C, Annesi-Maesano I, Cecchi L, Biagioni B, Chung F, D'Amato G, Damialis A, Del Giacco S, De Las Vecillas L, Dominguez-Ortega J, Galàn C, Gilles S, Giovannini M, Holgate S, Jeebhay M, Nadeau K, Papadopoulos N, Quirce S, Sastre J, Traidl-Hoffmann C, Walusiak-Skorupa J, Sousa-Pinto B, Salazar J, Rodríguez-Tanta LY, Cantero Y, Montesinos-Guevara C, Song Y, Alvarado-Gamarra G, Sola I, Alonso-Coello P, Nieto-Gutierrez W, Jutel M, Akdis CA. The impact of indoor pollution on asthma-related outcomes: A systematic review for the EAACI guidelines on environmental science for allergic diseases and asthma. Allergy. 2024 Jul;79(7):1761-1788.

7. Mendell MJ, Mirer AG, Cheung K, Tong M, Douwes J. Respiratory and allergic health effects of dampness, mold, and dampness-related agents: a review of the epidemiologic evidence. Environ Health Perspect. 2011 Jun;119(6):748-56.

8. Quansah R, Jaakkola MS, Hugg TT, Heikkinen SA, Jaakkola JJ. Residential dampness and molds and the risk of developing asthma: a systematic review and meta-analysis. PLoS One 2012;7(11): e47526.

9. Jaakkola JJ, Hwang BF, Jaakkola N. Home dampness and molds, parental atopy, and asthma in childhood: a six-year population-based cohort study. Environ Health Perspect 2005;113(3):357-61

10. Pekkanen J, Hyvärinen A, Haverinen-Shaughnessy U, Korppi M, Putus T, Nevalainen A: Moisture damage and childhood asthma: a population-based incident case-control study. Eur Respir J 2007;29:509-515.

11. Karvonen AM, Hyvärinen A, Korppi M, Haverinen-Shaughnessy U, Renz H, Pfefferle PI, Remes S, Genuneit J, Pekkanen J. Moisture Damage and Asthma: A Birth Cohort Study. Pediatrics 2015;135(3):e598-606.

12. Fisk WJ, Chan WR, Johnson AL. Does dampness and mold in schools affect health? Results of a meta-analysis. Indoor Air. 2019 Nov;29(6):895-902

13. Annila PJ, Lahdensivu J, Suonketo J, Pentti M, VinhaJ. Need to repair moisture- and mould damage in different structures in Finnish public buildings. Journal of Building Engineering 2018;16:72-78.

14. Annila P. Detecting Moisture and Mould Damage in Finnish Public Buildings. PhD thesis. Tampere University Dissertations 612, 2022.

15. Pitkäranta M (editor) 2016. Rakennuksen kosteus- ja sisäilmatekninen kuntotutkimus (Building moisture and indoor air quality assessment). Ministry of the Environment. Department of the Built Environment. Helsinki. 234 p. Available:http://urn.fi/URN:ISBN:978-952-11-4626-8. (Abstract in English)

16. Viljanen M, Kettunen AV, Kauriinvaha E, Bergman J, Laamanen P, Nevalainen A, Hyvärinen A, Mecklin T. Kosteus- ja homevaurioituneen rakennuksen kuntotutkimus (Condition investigation of moisture and mould damaged buildings). Ympäristöopas 28. Rakennustieto, Tampere 1997.

17. Mendell and Adams. The challenge for microbial measurements in buildings. Indoor Air 2019;(4):523-526.
