# Supplementary material for: Inspection confirmed mold damage in schools and new use of drugs for airway obstruction: A cohort study
Source: PLoS One. 2025 Oct 8;20(10):e0333486. doi: 10.1371/journal.pone.0333486 (PMC12507237; doi:10.1371/journal.pone.0333486)
Supplement: S2 Fig — (DOCX) [file pone.0333486.s002.docx]

S2 Figure. Graphical presentation of the design. Study population was based on list of students in February 2004. Their typical years spent in the school is marked with colors and follow-up until end of 2019 with a dashed line. Preschool year is marked separately, as not all buildings had preschool classes.
