# Supplementary material for: Inspection confirmed mold damage in schools and new use of drugs for airway obstruction: A cohort study
Source: PLoS One. 2025 Oct 8;20(10):e0333486. doi: 10.1371/journal.pone.0333486 (PMC12507237; doi:10.1371/journal.pone.0333486)
Supplement: S3 Table — Analyses limited to primary school students, whose home address did not change during primary school. (DOCX) [file pone.0333486.s003.docx]

| S3 Table. Association of mold damage in the school building with new asthma and | | | | | | | | | | |
| --- | --- | --- | --- | --- | --- | --- | --- | --- | --- | --- |
| new use of drugs for obstructive airways diseases using two different adjustments. | | | | | | | | | | |
| Analyses limited to primary school students, whose home address did not change during primary school. | | | | | | | | | | |
| Also four primary schools (8 buildings) removed, as school classes located in different buildings. | | | | | | | | | | |
|  |  |  |  |  |  |  |  |  |  |  |
| Mold damage | N of buildings | students at risk | n (%) of cases | Model 1*^a^* hazard ratios (95% CI) | Model 2*^b^* hazard ratios (95% CI) |  |  |  |  |  |
|  |  |  |  |  |  |  |  |  |  |  |
|  | *Development of new asthma*^c^ | | |  |  |  |  |  |  |  |
| No or small | 6 | 824 | 30 (3.6 %) | 1 | 1 |  |  |  |  |  |
| Limited | 13 | 1876 | 70 (3.7 %) | 1.01 (0.63, 1.62) | 0.90 (0.52, 1.58) |  |  |  |  |  |
| Wide | 33 | 5056 | 183 (3.6 %) | 0.98 (0.64, 1.51) | 0.85 (0.50, 1.45) |  |  |  |  |  |
| Very wide | 28 | 5051 | 150 (3.0 %) | 0.80 (0.51, 1.23) | 0.68 (0.39, 1.20) |  |  |  |  |  |
|  | *Development of new use of drugs for obstructive airways disease*^d^ | | | | | | | | | |
| No or small | 6 | 724 | 71 (9.8 %) | 1 | 1 |  |  |  |  |  |
| Limited | 13 | 1676 | 210 (12.5 %) | 1.28 (0.97, 1.67) | 1.37 (1.01, 1.86) |  |  |  |  |  |
| Wide | 33 | 4520 | 528 (11.7 %) | 1.18 (0.92, 1.51) | 1.26 (0.94, 1.69) |  |  |  |  |  |
| Very wide | 28 | 4514 | 517 (11.5 %9 | 1.16 (0.90, 1.48) | 1.24 (0.92, 1.69) |  |  |  |  |  |

*^a^*Model 1 adjusted for student’s age and sex.

*^b^*Model 2 adjusted in addition for year of construction, year of detection of moisture damage, mother's social class, mother’s language, maternal smoking during pregnancy, caesarean section, birth weight below 2500g, gestational age <36 weeks, and not having older siblings.

*^c^*Asthma defined based on 2 year use of inhaled corticosteroids.

*^d^*Use of drugs for obstructive airways diseases defined based on two prescriptions for any R03 drug within one year
