# Supplementary material for: Inspection confirmed mold damage in schools and new use of drugs for airway obstruction: A cohort study
Source: PLoS One. 2025 Oct 8;20(10):e0333486. doi: 10.1371/journal.pone.0333486 (PMC12507237; doi:10.1371/journal.pone.0333486)
Supplement: S4 Table — (DOCX) [file pone.0333486.s004.docx]

S4 Table. Association of mold damage in the school building with new asthma and new use of drugs for obstructive airways diseases using two different adjustments, separating the follow-up into periods during and after the school years. Analyses limited to primary school students only.

| Mold damage | N of buildings | students at risk | n (%) of cases | Model 1*^a^* hazard ratios (95% CI) | Model 2*^b^* hazard ratios (95% CI) |  |  |  |  |  |
| --- | --- | --- | --- | --- | --- | --- | --- | --- | --- | --- |
|  |  |  |  |  |  |  |  |  |  |  |
|  | *New asthma*^c^*: observed during the time the student was studying in the school* | | | | | | | | | |
| No or small | 9 | 1870 | 27 (1.4 %) | 1 | 1 |  |  |  |  |  |
| Limited | 14 | 3288 | 66 (2.0 %) | 1.31 (0.81, 2.13) | 1.25 (0.69, 2.28) |  |  |  |  |  |
| Wide | 34 | 8098 | 147 (1.8 %) | 1.15 (0.74, 1.79) | 1.09 (0.62, 1.94) |  |  |  |  |  |
| Very wide | 31 | 8954 | 120 (1.3 %) | 0.85 (0.55, 1.34) | 0.78 (0.43, 1.44) |  |  |  |  |  |
|  | *after the school years* | | |  |  |  |  |  |  |  |
| No or small | 9 | 1843 | 29 (1.6 %) | 1 | 1 |  |  |  |  |  |
| Limited | 14 | 3222 | 60 (1.9 %) | 1.17 (0.74, 1.85) | 0.99 (0.56, 1.75) |  |  |  |  |  |
| Wide | 34 | 7951 | 153 (1.9 %) | 1.23 (0.81, 1.85) | 1.04 (0.61, 1.77) |  |  |  |  |  |
| Very wide | 31 | 8834 | 164 (1.9 %) | 1.18 (0.78, 1.77) | 1.02 (0.58, 1.79) |  |  |  |  |  |
|  |  |  |  |  |  |  |  |  |  |  |
|  | *New use of R03 drug*^d^*: observed during the time the student was studying in the school* | | | | | | | | | |
| No or small | 9 | 1643 | 57 (3.5 %) | 1 | 1 |  |  |  |  |  |
| Limited | 14 | 2936 | 140 (4.8 %) | 1.30 (0.95, 1.79) | 1.40 (0.95, 2.04) |  |  |  |  |  |
| Wide | 34 | 7277 | 337 (4.6 %) | 1.21 (0.90, 1.61) | 1.30 (0.91, 1.87) |  |  |  |  |  |
| Very wide | 31 | 7984 | 370 (4.6 %) | 1.22 (0.91, 1.63) | 1.32 (0.91, 1.93) |  |  |  |  |  |
|  | *after the school years* | | |  |  |  |  |  |  |  |
| No or small | 9 | 1586 | 109 (6.9 %) | 1 | 1 |  |  |  |  |  |
| Limited | 14 | 2796 | 230 (8.2 %) | 1.21 (0.96, 1.52) | 1.15 (0.88, 1.52) |  |  |  |  |  |
| Wide | 34 | 6940 | 553 (8.0 %) | 1.19 (0.97, 1.47) | 1.13 (0.87, 1.46) |  |  |  |  |  |
| Very wide | 31 | 7614 | 586 (7.7 %) | 1.14 (0.93, 1.40) | 1.08 (0.83, 1.42) |  |  |  |  |  |

*^a^*Model 1 adjusted for student’s age and sex.

*^b^*Model 2 adjusted in addition for year of construction, year of detection of moisture damage, mother's social class, mother’s language, maternal smoking during pregnancy, caesarean section, birth weight below 2500g, gestational age <36 weeks, and not having older siblings.

*^c^*Asthma defined based on 2 year use of inhaled corticosteroids.

*^d^*Use of drugs for obstructive airways diseases defined based on two prescriptions for any R03 drug within one year
