# Supplementary material for: Inspection confirmed mold damage in schools and new use of drugs for airway obstruction: A cohort study
Source: PLoS One. 2025 Oct 8;20(10):e0333486. doi: 10.1371/journal.pone.0333486 (PMC12507237; doi:10.1371/journal.pone.0333486)
Supplement: S5 Fig — (DOCX) [file pone.0333486.s005.docx]

S5 Fig. Year of construction of the school building by extent of mold damage
